# Force-induced recruitment of cten along keratin network in epithelial cells

**DOI:** 10.1073/pnas.1911865116

**Published:** 2019-09-16

**Authors:** Joleen S. Cheah, Kyle A. Jacobs, Volkmar Heinrich, Su Hao Lo, Soichiro Yamada

**Affiliations:** ^a^Department of Biomedical Engineering, University of California, Davis, CA 95616;; ^b^Department of Biochemistry and Molecular Medicine, School of Medicine, University of California, Davis, CA 95817

**Keywords:** cytoskeleton, mechanotransduction, keratin, tensin, simple epithelia

## Abstract

The cytoskeleton provides structural integrity to cells and serves as a key component in mechanotransduction. Tensins are thought to provide a force-bearing linkage between integrins and the actin cytoskeleton; yet, direct evidence of tensin’s role in mechanotransduction is lacking. We here report that local force application to epithelial cells using a micrometer-sized needle leads to rapid accumulation of cten (tensin 4), but not tensin 1, along a fibrous intracellular network. Surprisingly, cten-positive fibers are not actin fibers; instead, these fibers are keratin intermediate filaments. The dissociation of cten from tension-free keratin fibers depends on the duration of cell stretch, demonstrating that the external force favors maturation of cten−keratin network interactions over time and that keratin fibers retain remarkable structural memory of a cell’s force-bearing state. These results establish the keratin network as an integral part of force-sensing elements recruiting distinct proteins like cten and suggest the existence of a mechanotransduction pathway via keratin network.

Physical integrity of epithelial tissue is established and maintained by the cytoskeletal network that integrates neighboring cells and the extracellular matrix. The actin cytoskeleton, together with actin-binding proteins and adhesive junctions, has been shown to respond to physical forces. The tensin family consists of 4 members that all reside at focal adhesions and have a major role in linking integrins to the actin cytoskeleton. As the name implies, tensin 1 was originally thought to maintain “tension” between the actin cytoskeleton and adhesive contacts (1). Tensins bind to β-integrins through their phosphotyrosine (pTyr) binding domains, and to pTyr-containing proteins, including Axl, Src, Fak, p130cas, paxillin, and Rho GAP DLC1 (deleted in liver cancer), via SH2 (Src homology 2) domains (1, 2). These interactions provide molecular linkages between integrin receptors and the cytoskeleton and mediate signaling transduction. Altogether, they contribute to actin cytoskeleton organization, focal adhesion dis/assembly, cell adhesion, migration, proliferation, and survival (1–3). Yet, how physical forces regulate and alter tensins’ functions has not been determined.

To test this force-dependent regulation of tensin dynamics, tensin 1, a prototypical tensin, and cten (tensin 4), the shortest member of the tensin family, were green fluorescent protein (GFP)-tagged and expressed in epithelial [Madin−Darby canine kidney (MDCK)] cells. A micrometer-sized glass needle tip was used to poke or press onto a sacrificial cell in the epithelial cell colony, then pulled away from the colony to apply mechanical strain onto the adjacent cells (Fig. 1*A* and Movie S1). While subcellular localization of tensin 1 remained similar to unstretched cells (Fig. 1*B* and Movie S2), cten rapidly accumulated along fibrous structures (Fig. 1*A* and Movie S1) with as little as <10% total cell strain (Fig. 1*C*). Interestingly, tensin 1 and cten intensity at focal adhesions did not change as cells were stretched (Fig. 1 *A* and *B*, arrowheads, and Fig. 1*D*), suggesting that the amount of tensins at focal adhesions is insensitive to cell stretch. The cten-positive fibers often terminated at cell−cell contacts with the sacrificial cells, while the other ends of these fibers frequently ended at the center of cells or at the opposite cell−cell contacts (Fig. 1*A*; see also Fig. 1 *F* and *H*). The cten-positive fibers often had the highest intensity in the cell interior, indicating robust cten recruitment along the fibers (Fig. 1*E*; a ratio of >1 indicates that cten accumulation in subcellular regions is greater for stretched cells than for prestretch cells). This unique cten accumulation is not limited to MDCK cells. GFP-tagged cten expressed in mammary epithelial (184B) cells also rapidly accumulated along fibrous structures (Fig. 1*E* and Movie S3).

**Fig. 1.**
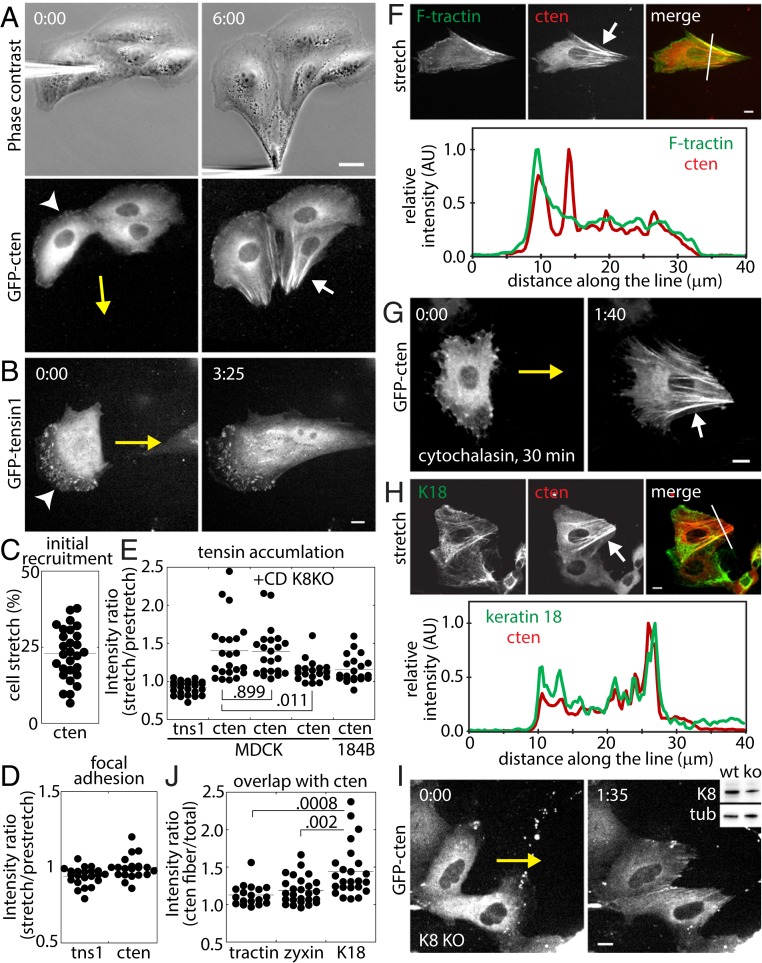
Force-induced cten accumulation occurs along keratin fibers. (*A*) The cten dynamics under tension. (*B*) Tensin 1 dynamics under tension. (*C*) The extent of cell stretch (percent) at the first appearance of cten-positive fibers (*n* = 28). (*D*) The fluorescence intensity ratio (stretch/prestretch) of tensins at focal adhesions; *n* = 22 (tensin 1) and 20 (cten). (*E*) Quantification of cten accumulation; *n* = 24 (tensin 1), 22 (cten), 22 (CD), 18 (K8KO), and 21 (184B). The cten intensities were quantified at average cell stretch values of 85 ± 52% (cten), 96 ± 36% (+CD), and 71 ± 28% (K8KO). (*F*) Colocalization of F-tractin–GFP and tomato–cten, and the intensity scan along the line shown in the merged image. (*G*) Cytochalasin D at 2 μM has no effect on cten accumulation. (*H*) Colocalization of mEmerald–keratin 18 and tomato-cten. (*I*) The cten accumulation is reduced in keratin 8-deficient cells. (*Inset*) Western blot of keratin 8 and tubulin for wild-type (wt) and partial knockout (ko) cells. (*J*) Relative localization of F-tractin, zyxin, and keratin 18 at cten-positive fibers; *n* = 18 (F-tractin), 26 (zyxin), and 24 (K18). In all images, yellow arrows denote the microneedle movement, white arrowheads point to focal adhesions, and the white arrow points to tensin-positive fibrous structures. (Scale bar, 20 μm in *A* and 10 μm in others.) In *A*, *B*, *G* and *I*, time in minutes:seconds. All analysis includes at least 2 independent repeats.

To determine the identity of cten-positive fibers, the epithelial cells were cotransfected with F-tractin, an actin binding domain from inositol 1,4,5-triphosphate 3-kinase A (4) or zyxin, a focal adhesion protein known to localize to force-bearing actin stress fibers (5, 6), and cten. As cells were stretched with a microneedle, preexisting F-tractin–positive actin fibers elongated in the stretch direction (Fig. 1*F* and Movie S4) and zyxin accumulated along force-bearing actin fibers (Movie S5). Interestingly, however, cten did not exclusively accumulate along actin fibers or zyxin-positive fibers (Fig. 1*J*). For example, the large actin bundles along the cell periphery colocalized with cten, but no obvious fibrous actin network colocalized with other numerous cten-positive fibers in the central region of cells (Fig. 1*F*). In addition, cten was still recruited to fibrous structures in the presence of cytochalasin D (Fig. 1 *E* and *G* and Movie S6), suggesting that cten-positive fibers are not actin bundles.

The cten-positive fibers often terminated at cell−cell contacts, resembling the organization of the keratin intermediate filament network. MDCK cells express keratin 7, 8, 18, and 19, a set of keratins often expressed in simple epithelia (7). Under tension from the microneedle, the keratin network oriented along the stretch direction similar to the actin filament network (Fig. 1*H* and Movie S7). More notably, these keratin 18 fibers, both small and large, colocalized with cten-positive fibers (Fig. 1 *H* and *J*). In keratin 8 (a natural partner of keratin 18) deficient cells, cten accumulation along the fibers was significantly reduced (Fig. 1 *E* and *I* and Movie S8) although not completely eliminated, likely due to residual keratin fibers present in these cells. Together, these results suggest that cten is recruited to keratin fibers in a force-dependent manner.

In rare cases, some keratin fibers tore from excessive strain applied by a microneedle, and cten remained localized along broken keratin fibers despite the release in tension (Movie S9). To systematically analyze cten dissociation after relieving the tension along keratin fibers, GFP–cten expressing cells were stretched and released, and then the decay in cten intensity along the fibers was quantified (Fig. 2 *A* and *B* and Movie S10). The decrease in cten intensity was interpreted as cten disassembly from the keratin network. This disassembly rate of cten did not correlate with the degree of cell stretch quantified by the maximum cell stretch level (Fig. 2*C*), but the disassembly rate of cten inversely correlated with the duration of stretch (Fig. 2*D*). These results suggest that prolonged application of stretch may alter the conformation of keratin fibers, thereby strengthening the interactions between keratin fibers and cten, and that keratin fibers retain structural memory of a force-bearing state even after tension release.

**Fig. 2.**
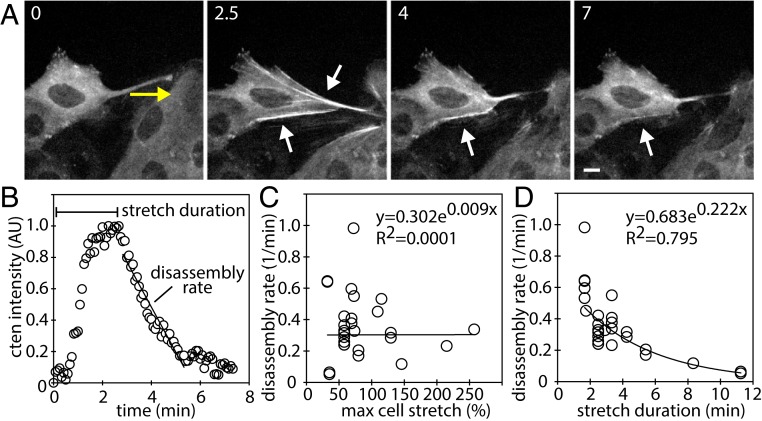
History-dependent cten stability along keratin network. (*A*) GFP–cten expressing cells were stretched (0 min to 2.5 min) then relaxed (4 and 7 min). Yellow arrow denotes the microneedle movement, and the white arrows point to tensin-positive fibrous structures. Time in minutes. (Scale bar, 10 μm.) (*B*) The cten dynamics along the fibers as the cell stretches and recoils. (*C*) The cten disassembly rate did not correlate with maximum cell stretch. (*D*) The cten disassembly rate decreased as cells were stretched over longer periods of time; *n* = 28 with 2 independent repeats.

Collectively, our analysis establishes the keratin network as an integral part of force-sensing elements similar to actin filaments (8, 9). Unlike actin and microtubule filaments, keratin networks are capable of withstanding larger strains (10, 11), providing a greater dynamic force-sensing range than actin filaments. The application of strain to single keratin filaments increases their length while reducing the apparent width of the filaments (11), suggesting that keratin subunits within the fibers can slide past each other and/or undergo partial unfolding of α-helical structures (12). One possibility for the strengthening of cten−keratin interactions upon cell stretch (Fig. 2*D*) is that this structural change in keratin fibers may expose cryptic cten-binding sites or alter their conformation for more favorable cten−keratin network interactions. This type of force-dependent regulation of cytoskeletal network has been previously observed in actin filaments. For example, tensile force along actin filaments can prevent severing by cofilin (13), increase formin-mediated actin polymerization (14), and increase the affinity of myosin II to actin filaments (15). Alternatively, cten may recognize other keratin filament binding proteins in a force-dependent manner. Our observation demonstrates the force-sensitive and history-dependent nature of protein interactions along keratin fibers. With cten often up-regulated in cancer (3) and keratins emerging as a key factor in collective cell migration (16–18), this cten−keratin network force-dependent interaction may play a critical role in cancer progression.

## Materials and Methods

### Cell Lines and Reagents.

MDCK GII and 184B (human mammary epithelial cells) cells were cultured in Dulbecco’s modified Eagle’s medium (Gibco) supplemented with 10% fetal bovine serum (Atlanta Biological) and HuMEC (human mammary epithelial cell) ready media (Gibco), respectively. Cells were either transiently or stably transfected with plasmids using Lipofectamine 3000 (Invitrogen) or jetOPTIMUS (Polyplus). Tensin-1 and cten plasmids were previously described (19). GFP-tagged F-tractin (58473) and mEmerald-tagged keratin 18 (54134) plasmids were purchased from Addgene. MDCK cells deficient in keratin 8 were generated using a CRISPR/Cas9 plasmid encoding the canine keratin 8-specific guide RNA (gtgatgctccccacgacccc), which resulted in partial knockout cells with a reduced level of keratin 8 (47% of the wild-type cells—Fig. 1*I*). Anti-keratin 8/18 (C51) and anti-α-tubulin (DM1A) antibodies were purchased from Cell Signaling Technology.

### Microscopy and Image Analysis.

Cells were imaged using a Zeiss AxioObserver equipped with a Yokogawa CSU-10 spinning disk confocal system, 40× objective, 488- and 561-nm solid-state lasers, a Photometrics CoolSNAP HQ2 camera, and Slidebook software (Intelligent Imaging Innovations). For live-cell imaging, the temperature was set to 37 °C. Intensities at focal adhesions were measured before stretch (prestretch) and at the maximum stretch (stretch), then plotted as ratios (Fig. 1*D*). Initial cten recruitment to fibers was detected manually, and the cell length along the stretch axis before (L_o_) and after (L) stretch was measured to calculate the degree of cell stretch (L/L_o_ − 1; Fig. 1*C*). Since the intensity of cten fibers often exceeded the cytoplasmic cten intensity, the ratio of the brightest regions (top 1% of the intensity) from prestretch and at maximum stretch was used as a proxy for cten fiber formation (Fig. 1*E*). Colocalization of cten with F-tractin, zyxin, and keratin 18 was quantified based on the relative intensities of the respective proteins at cten-positive fibers and cytoplasm (Fig. 1*J*). Images and data were analyzed using ImageJ, Microsoft Excel, and KaleidaGraph.

### Microneedle Cell Stretching Analysis.

Custom drawn needles were fabricated using P-97 Micropipette puller (Sutter Instrument). The microneedle was attached to a 3D micromanipulator (Physik Instrumente) controlled with a gaming joystick and custom-written software. The microneedle was carefully placed on neighboring sacrificial cells, then slowly moved away from the cells of interest, and strain was transmitted via cell−cell contacts. Note that none of the cells analyzed came in direct contact with the microneedle.

## Supplementary Material

Supplementary File

Supplementary File

Supplementary File

Supplementary File

Supplementary File

Supplementary File

Supplementary File

Supplementary File

Supplementary File

Supplementary File
